# What Every Woman Knows

**Published:** 1920-02-14

**Authors:** 


### What Every Woman Knows.

DR. ELIZABETH SLOAN CHESSER certainly gained the unanimous approval of her sex when, in her exceedingly attractive lecture at the Institute of Hygiene, she called attention to the beneficial effect of new clothes upon the human woman. Dress, she says, has a tremendous influence on mentality. The nervous depressed type of woman responds particularly well to the stimulus of beautiful clothes. When people are mentally and spiritually at a low ebb they should try what buying something nice will do—even if it means selling old so-called ornaments of which they have grown weary. A new hat, she declares, may have a greater therapeutic stimulative value than any tonic bought at a chemists.

It can be imagined how valuable Dr. Chesser's dicta will become. It is medical testimony to what every woman knows. The chemist must look upon the costumier as a rival!

Dr. Chesser has a gift for expressing the principles of hygiene in a bright and forceful manner. She stings her audience into attention with (as an instance) the suggestion that many matrimonial differences are caused through tight collars. She then elaborates her statement by a logical disquisition on the evils of tight clothing generally—how it obstructs circulation, causes varicose veins, cold feet, chilblains, headache, and bad temper. It is all perfectly reasonable, though few could state the case in a way which obtains so much publicity to the propaganda of hygiene. Children, says Dr. Chesser, should be kept warm with as few garments as possible.

Clothing should be loose, and the neck might be properly exposed, but never the apices of the lungs. Dainty shoes and stockings were quite out of place in London, and were responsible for many cases of influenza and pneumonia. Woollen stockings and stout-soled shoes were the proper wear. Women should favour more changes of clothes, and send their garments more often to the cleaners. If a white garment got filthy in a couple of days, what must a coloured one be like in a couple of months? Clothing should be light, and, to allow moisture to escape, should be porous. Veils, she thought, were dirty, and did not keep out germs. Her ideal of a fit-out for a boy was woollen combinations, light or heavy, always, with shirt, knickerbockers, and jersey, and the same for girls with the addition of a skirt.

